# Spectrum and epidemiology of rare diseases in a Chinese natural population of 14.31 million residents, 2012–2023

**DOI:** 10.1186/s13023-025-03933-8

**Published:** 2025-08-07

**Authors:** Ming-Jia Li, Qi Li, Miao-Miao Zhao, Hanna Kim, Ruo-Qi Feng, Mo-Ning Guo, Jian-Peng heng, Jin-Kui Yang, Chang Liu

**Affiliations:** 1 Department of Endocrinology, Beijing Tongren Hospital, Capital Medical University, Beijing, 100730 China; 2 Beijing Diabetes Institute, Beijing Tongren Hospital, Capital Medical University, Beijing, 100730 China; 3 Beijing Municipal Health Commission Information Center, Beijing, 100034 China

**Keywords:** Rare disease, Population-based study, Incidence, Prevalence, Wilson’s disease, Generalized myasthenia gravis, ANCA-associated vasculitis, Malignant melanoma

## Abstract

**Background:**

Rare diseases, though individually uncommon, collectively affect a significant portion of the population. However, their epidemiology in China remains underexplored. A population-based rare disease registry comprising 14.31 million individuals was conducted between 2012 and 2023 by the Beijing Municipal Health Big Data and Policy Research Center. Rare disease cases were identified via ICD-10 codes mapped to China’s national rare disease lists (2018 and 2023) and international databases. Age-standardized incidence rates (ASIR) were calculated per 100,000 person-years with 95% confidence intervals.

**Results:**

Our analysis identified 12,371 rare disease cases, with the overall ASIR increasing from 6.109 in 2012 to 7.394 in 2023. Rare neurologic diseases accounted for 52.12% of cases, followed by systemic and rheumatologic diseases (16.89%) and rare neoplastic diseases (9.99%). The most frequently diagnosed rare diseases included generalized myasthenia gravis, ANCA-associated vasculitis, and malignant melanoma. Significant sex-based differences were observed, with female patients more affected by systemic and rheumatologic conditions, while male patients showed a higher incidence of respiratory disorders. Pediatric patients predominantly presented with inborn errors of metabolism and rare immune diseases. Comparisons with global data revealed notable disparities, such as a higher prevalence of Wilson’s disease and a lower incidence of amyotrophic lateral sclerosis (ALS) in China.

**Conclusions:**

This study represents the first large-scale, population-based analysis of rare diseases in China, revealing distinct epidemiological patterns. These findings underscore the critical need for healthcare policies that address the unique challenges posed by rare diseases in China.

**Supplementary Information:**

The online version contains supplementary material available at 10.1186/s13023-025-03933-8.

## Background

Rare diseases, also known as orphan diseases, are conditions with a low prevalence in the general population. While each rare disease affects a small number of individuals, collectively they have a profound global impact, touching the lives of numerous families and communities [1]. To date, over 7,000 rare diseases have been identified worldwide, affecting approximately 3.5–5.9% of the global population [2]. In recent years, rare diseases have garnered increasing attention in China, leading to significant milestones such as the establishment of the first national rare disease catalog in 2018 [3], encompassing 121 conditions, and the release of a second national catalog in 2023 [4], listing an additional 86 rare diseases. Despite these efforts, there remains a substantial gap in understanding the epidemiology and spectrum of rare diseases in the Chinese population.

China defines rare diseases as conditions meeting at least one of three criteria: an incidence among newborns of less than 1 in 10,000, a prevalence of less than 1 in 10,000, and an affected population of fewer than 140,000 individuals [5]. This definition, along with the rare diseases list, has now been officially adopted nationwide.

As significant advancements are being made in the development of new therapies for rare diseases, there is an urgent need to gather robust epidemiological data on these conditions within the general population. Gene therapies, such as *Zolgensma* (Onasemnogene abeparvovec-xioi) for spinal muscular atrophy [6, 7] and *Luxturna* (voretigene neparvovec-rzyl) for inherited retinal dystrophy [8], represent breakthroughs in genetic medicine. Enzyme replacement therapies (ERTs), like *Cerezyme* (Imiglucerase) for Gaucher disease and *Aldurazyme (*laronidase) for mucopolysaccharidosis, have improved clinical outcomes and quality of life for patients with lysosomal storage disorders [9]. Additionally, small molecule drugs, such as *Kalydeco (*ivacaftor) for cystic fibrosis, target specific genetic mutations, offering more effective treatments [10]. Comprehensive epidemiological data not only help identify populations in need of care but also inform healthcare resource allocation and policy development. With the continuous emergence of novel therapies, accurate and updated data will be crucial for ensuring that these treatments reach the right patients and for optimizing healthcare planning [11, 12].

The rarity of these diseases presents significant challenges, including delayed diagnosis, limited treatment options, and insufficient research funding. In region with established rare disease databases, such as Europe (e.g., Orphadata [13]), comprehensive epidemiological studies have significantly improved disease management and policymaking [14]. However, similar large-scale population-based studies are scarce in China, partly due to the lack of systematically collected long-term data.

This study leverages a 12-year dataset of 14.31 permanent registered residents in the Great Beijing Area to analyze the spectrum of rare diseases in the Chinese natural population. By utilizing ICD-10 codes for case identification, we compare the findings with both China’s national rare disease list and the international catalog of rare diseases. This approach aims to provide a comprehensive overview of rare disease distribution in China and to explore potential differences with other populations globally.

Understanding the spectrum of rare diseases in China is critical for improving diagnostic accuracy, optimizing resource allocation, and informing health policy. Furthermore, this study contributes valuable data to the global rare disease research community, addressing the underrepresentation of Asian populations in existing literature.

## Methods

### Study design and participants

This retrospective, population-based study included 14.31 million permanent registered residents of the Great Beijing Area from 2012 to 2023. The study population was derived from the Beijing Municipal Health Commission Information Center (BMHCIC), which aggregates diagnostic records from all hospitals authorized to diagnose rare diseases across 16 districts (8 urban and 8 suburban) in the Great Beijing Area. We excluded migrant populations and active military personnel to ensure accurate residency data. Demographic details—including age, sex, and admission dates—were obtained for each patient.

### Data sources

Data were extracted from the BMHCIC database, which compiles diagnostic records from all designated hospitals in the Great Beijing Area. The dataset covers the period from 2012 to 2023 and includes ICD-10 codes for rare disease diagnoses, demographic information, and admission dates. Residency information was validated via integration with the Municipal Public Security Bureau’s household registration system.

### ICD-10 codes and case identification

Case identification employed a dual-validation framework integrating domestic and international standards. First, ICD-10 codes were systematically mapped to the First National List of Rare Diseases (2018) and the Second National List of Rare Diseases (2023) of China through standardized crosswalk protocols. Second, these codes underwent alignment with one of the major global repositories: Orphadata [15].

### Data linkage and validation

Residency status was validated through integration with the Municipal Public Security Bureau’s household registration database. Migrant populations and active military personnel were excluded. Incidence and prevalence calculations used annual registered resident counts as denominators. Using patients’ national identification numbers, we linked health records to residency data to accurately calculate the incidence of rare diseases among registered residents.

### Statistical analysis

Incidence rates of rare diseases were calculated by dividing the number of identified cases by the total permanent population at risk. Age-standardized rates were computed using the direct standardization method to allow comparisons with international data. Incidence rates were expressed per 100,000 person-years with 95% confidence intervals (CI) using Poisson approximations.

The spectrum of rare diseases was analyzed by Orphanet classification based on their affected organ systems and clinical manifestations. To assess differences between the Chinese population and international datasets, comparative analyses were conducted using data from Orphadata [16] and other published global reports.

Data analyses were performed using R (version 4.2.2). Statistical significance was defined as a two-sided *p*-value < 0.05.

## Results

### Study participants and overall incidence (2012–2023)

**Fig. 1:**
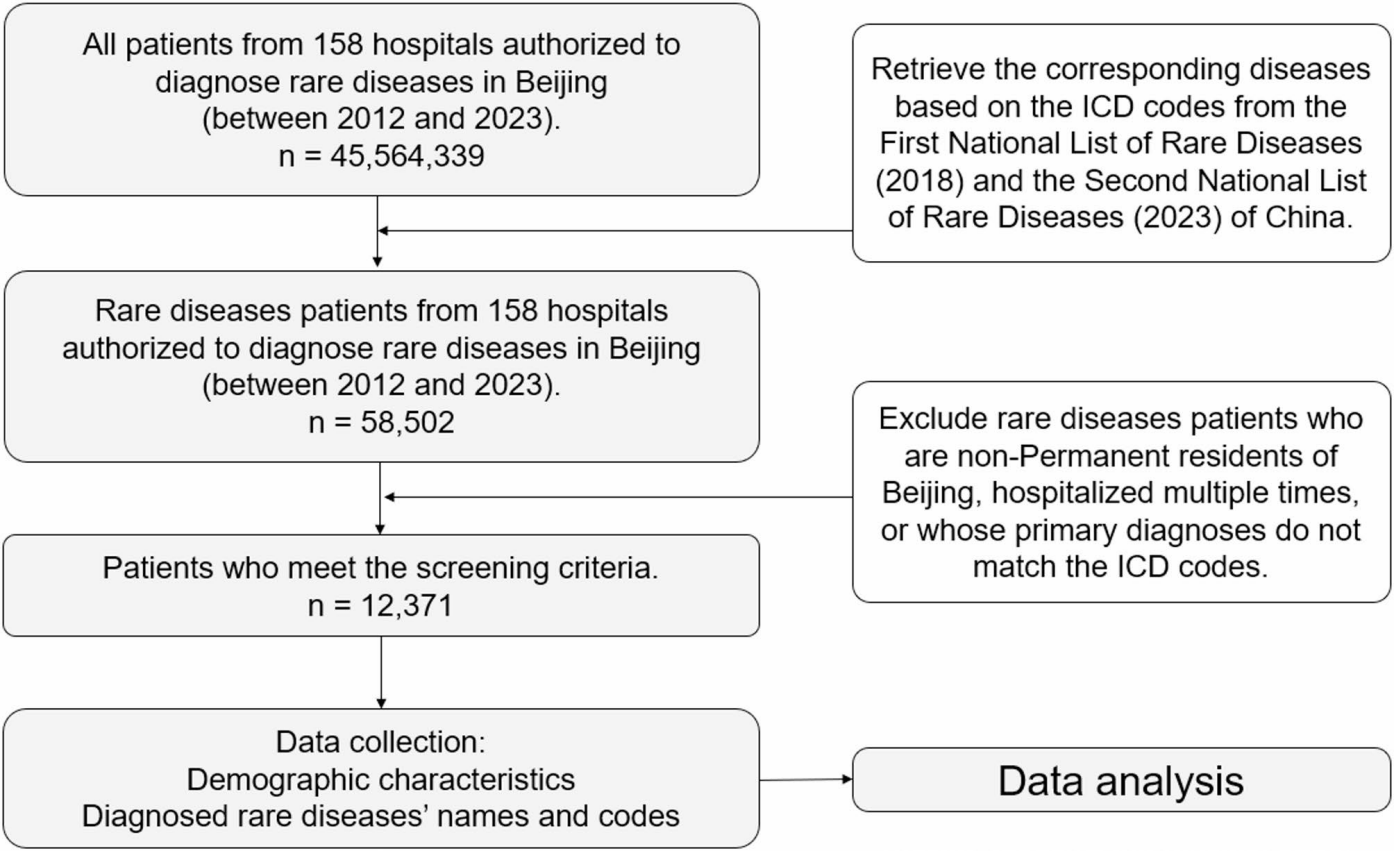
The flow chart of the rare disease cases

Between 2012 and 2023, we captured 12,371 newly diagnosed rare disease cases from all 158 authorized centers in the Great Beijing Area, covering 12.97 million residents in 2012 and 14.31 million in 2023 (Supplementary Table 1; Fig. 1). Of these cases, 6,381 (51.58%) were male and 5,990 (48.42%) were female. The mean age at diagnosis was 53.8 years (median 60.0), with 1,370 cases (11.07%) occurring in individuals aged ≤ 18 years (Table 1). The overall age-standardized incidence rate (ASIR) rose from 6.109 per 100,000 person-years (95% CI: 4.901–7.524) in 2012 to 7.394 (95% CI: 6.058–8.937) in 2023.

**Table 1:** Demographic characteristics and ASIR (2012, 2023) of the cohort by Orphanet classification

Orphanet classification	All Cases			≤ 18 years old Cases *n* (%)	Mean/Median age (years)	ASIR (95% CI)	
	*n* (%)	Men	Women			2012	2023
Total	12,371 (100.00)	6381	5990	1370 (100.00)	53.8 / 60.0	6.109 (4.901, 7.524)	7.394 (6.058, 8.937)
Rare neurologic disease	6448 (52.12)	3280	3168	640 (46.72)	52.8 / 58.0	2.767 (1.977, 3.769)	3.885 (2.935, 5.043)
Rare systemic & rheumatologic disease	2090 (16.89)	983	1107	26 (1.90)	67.7 / 69.0	1.093 (0.622, 1.780)	1.329 (0.802, 2.071)
Rare neoplastic disease	1236 (9.99)	594	642	7 (0.51)	62.4 / 65.0	0.827 (0.427, 1.447)	0.738 (0.364, 1.333)
Rare respiratory disease	442 (3.57)	319	123	64 (4.67)	57.2 / 66.0	0.069 (0.002, 0.385)	0.271 (0.072, 0.701)
Rare eye disease	439 (3.55)	192	247	9 (0.66)	51.5 / 53.0	0.411 (0.150, 0.899)	0.202 (0.040, 0.598)
Rare hematologic disease	362 (2.93)	216	146	61 (4.45)	46.0 / 50.5	0.297 (0.086, 0.739)	0.227 (0.051, 0.635)
Inborn errors of metabolism	314 (2.54)	190	124	254 (18.54)	11.0 / 1.0	0.180 (0.031, 0.565)	0.159 (0.023, 0.532)
Rare circulatory system disease	254 (2.05)	157	97	94 (6.86)	37.9 / 51.0	0.024 (0.000, 0.303)	0.143 (0.018, 0.508)
Rare gastroenterologic disease	248 (2.00)	157	91	15 (1.09)	49.1 / 50.0	0.184 (0.033, 0.571)	0.148 (0.020, 0.516)
Rare hepatic disease	192 (1.56)	107	85	86 (6.28)	24.3 / 22.5	0.089 (0.005, 0.419)	0.171 (0.028, 0.551)
Rare cardiac disease	180 (1.46)	113	67	30 (2.19)	43.6 / 48.0	0.119 (0.011, 0.469)	0.010 (0.000, 0.275)
Rare renal disease	75 (0.61)	37	38	20 (1.46)	31.9 / 33.0	0.019 (0.000, 0.295)	0.050 (0.000, 0.352)
Rare developmental defect	46 (0.37)	20	26	33 (2.41)	13.6 / 5.0	0.022 (0.000, 0.299)	0.044 (0.000, 0.341)
Rare bone disease	30 (0.24)	7	23	24 (1.75)	16.5 / 10.0	0.000 (0.000, 0.255)	0.000 (0.000, 0.255)
Rare skin disease	6 (0.05)	3	3	1 (0.07)	40.8 / 48.5	0.007 (0.000, 0.271)	0.008 (0.000, 0.272)
Rare endocrine disease	6 (0.05)	4	2	3 (0.22)	32.0 / 32.5	0.000 (0.000, 0.255)	0.010 (0.000, 0.275)
Rare immune disease	3 (0.02)	2	1	3 (0.22)	4.3 / 4.0	0.000 (0.000, 0.255)	0.000 (0.000, 0.255)

ASIR, age-standardized incidence rate, per 100,000 person-years (95%CI)

ASIR for all rare diseases are based on the standard population published in the 2023 China Statistical Yearbook

**Table 2:** Demographic characteristics and ASIR (2012,2023) of the top disease in each Orphanet classification of the cohort

Top 1 disease in each Orphanet classification	All Cases			≤ 18 years old Cases	Mean/Median age (years)	ASIR (95% CI)		
	*n*	Men	Women			2012		2023
Neurologic: Generalized myasthenia gravis	3310	1656	1654	220	58.3 / 63.0	1.532 (0.961, 2.316)		2.143 (1.456, 3.043)
Rheumatologic: ANCA-associated vasculitis	2025	949	1076	14	68.3 / 70.0	1.078 (0.611, 1.762)		1.275 (0.761, 2.005)
Neoplastic: Melanoma	1176	570	606	6	63.0 / 65.0	0.827 (0.427, 1.447)		0.720 (0.351, 1.309)
Respiratory: Idiopathic pulmonary fibrosis	286	232	54	0	71.8 / 72.0	0.011 (0.000, 0.278)		0.184 (0.033, 0.571)
Eye: Retinitis pigmentosa	439	192	247	9	51.5 / 53.0	0.411 (0.150, 0.899)		0.202 (0.040, 0.598)
Hematologic: Paroxysmal nocturnal hemoglobinuria	148	86	62	4	53.3 / 54.0	0.151 (0.021, 0.519)		0.081 (0.003, 0.406)
Metabolism: Methylmalonic acidemia without homocystinuria	128	70	58	115	6.8 / 2.0	0.056 (0.001, 0.362)		0.088 (0.004, 0.418)
Circulatory: Coronary artery ectasia	254	157	97	94	37.9 / 51.0	0.024 (0.000, 0.303)		0.143 (0.018, 0.508)
Gastroenterologic: Familial adenomatous polyposis	130	79	51	4	42.1 / 40.0	0.054 (0.001, 0.358)		0.065 (0.001, 0.379)
Hepatic: Wilson disease	192	107	85	86	24.3 / 22.5	0.089 (0.005, 0.419)		0.171 (0.028, 0.551)
Cardiac: Noncompaction of ventricular myocardium	166	110	56	27	43.3 / 47.5	0.093 (0.005, 0.425)		0.010 (0.000, 0.275)
Renal: Gitelman syndrome	75	37	38	20	31.9 / 33.0	0.019 (0.000, 0.295)		0.050 (0.000, 0.352)
Developmental: 21-hydroxylase deficiency	25	9	16	20	11.1 / 5.0	0.017 (0.000, 0.289)		0.000 (0.000, 0.255)
Bone: McCune-Albright Syndrome	30	7	23	24	16.5 / 10.0	0.000 (0.000, 0.255)		0.000 (0.000, 0.255)
Skin: Pustular psoriasis	6	3	3	1	40.8 / 48.5	0.007 (0.000, 0.271)		0.008 (0.000, 0.272)
Endocrine: Homozygous hypercholesterolemia	5	3	2	2	35.6 / 51.0	0.000 (0.000, 0.255)		0.000 (0.000, 0.255)
Immune: X-linked lymphoproliferative disease	2	2	0	2	4.5 / 4.5	0.000 (0.000, 0.255)		0.000 (0.000, 0.255)
**Top 5 disease in each Orphanet classification**								
Neurologic: Generalized myasthenia gravis	3310	1656	1654	220	58.3 / 63.0	1.532 (0.961, 2.316)		2.143 (1.456, 3.043)
Rheumatologic: ANCA-associated vasculitis	2025	949	1076	14	68.3 / 70.0	1.078 (0.611, 1.762)		1.275 (0.761, 2.005)
Neoplastic: Melanoma	1176	570	606	6	63.0 / 65.0	0.827 (0.427, 1.447)		0.720 (0.351, 1.309)
Neurologic: Multiple sclerosis	803	251	552	27	42.3 / 41.0	0.651 (0.303, 1.219)		0.571 (0.250, 1.114)
Neurologic: Amyotrophic lateral sclerosis	634	351	283	0	60.2 / 62.0	0.343 (0.110, 0.803)		0.428 (0.160, 0.921)

ASIR, age-standardized incidence rate, per 100,000 person-years (95%CI)

ASIR for all rare diseases are based on the standard population published in the 2023 China Statistical Yearbook

### Spectrum and incidence by disease category

**Fig. 2:**
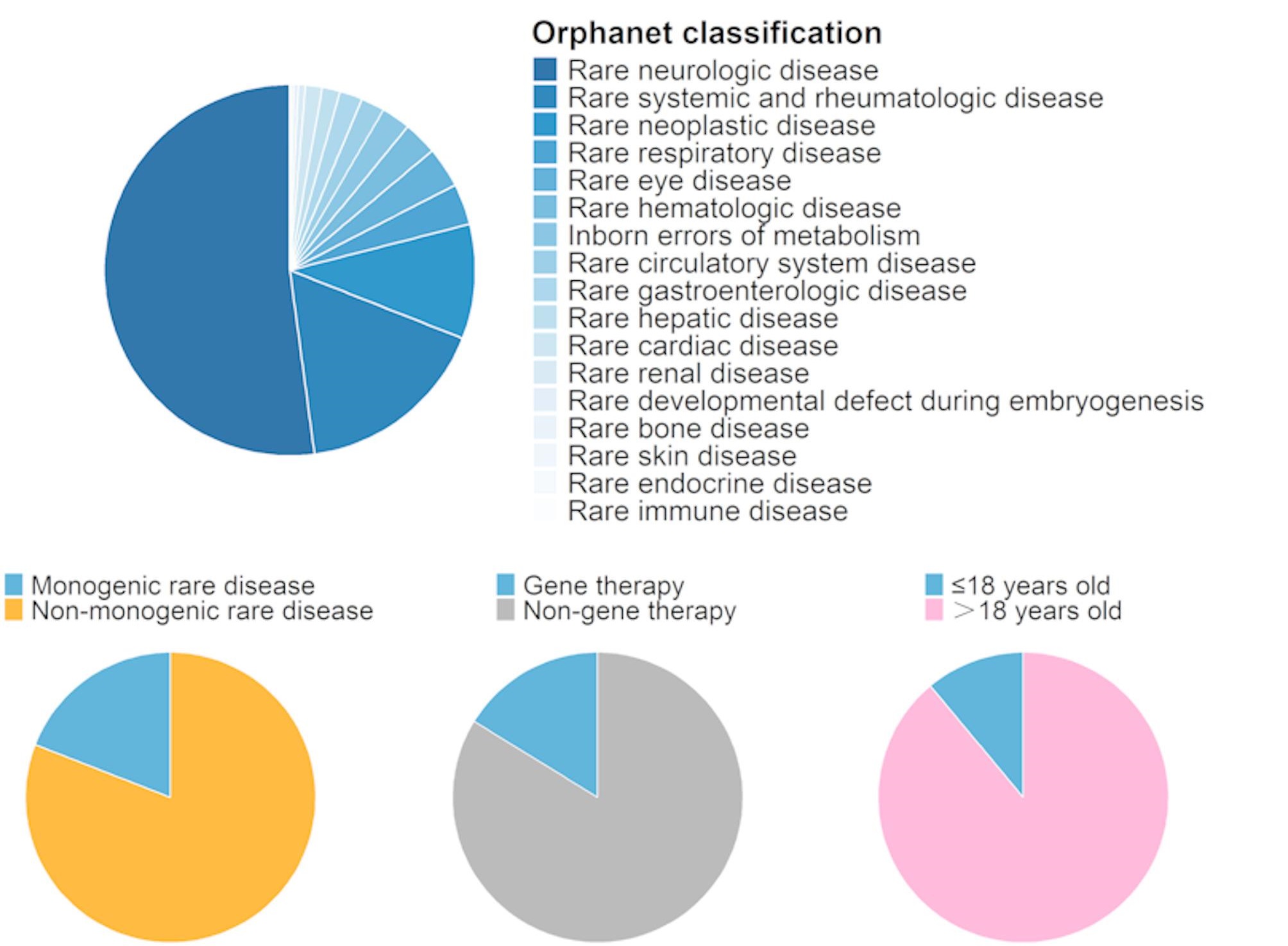
Distribution of rare diseases by Orphanet classification and other characteristics. (**A**) Proportions of rare disease groups based on Orphanet classification. (**B**) Comparison of monogenic and non-monogenic diseases. (**C**) Classification of rare diseases by treatment type (gene therapy vs. non-gene therapy). (**D**) Age distribution of rare diseases in population groups aged ≤ 18 years and > 18 years.

Using Orphanet classification [17–19], we first examined rare diseases by affected organ system (Supplementary Table 2; Fig. 2A). Rare neurologic diseases predominated, accounting for 52.12% of all cases and 46.72% of those ≤ 18 years. Systemic and rheumatologic diseases were second (16.89%), followed by neoplastic diseases (9.99%). Categories with very low incidence included skin diseases (0.05%), endocrine (0.05%), and immune diseases (0.02%) (Table 1; Fig. 2A). Over the 12 years, the ranking of ASIR by category remained stable: neurologic diseases had the highest ASIR—reaching 3.885 per 100,000 person-years (95% CI: 2.935–5.043) in 2023—while systemic/rheumatic diseases maintained an ASIR of 1.329 (95% CI: 0.802–2.071). Bone and immune diseases persisted at near-zero ASIRs (0.000; 95% CI: 0.000–0.255 in 2023).

Next, we classified diseases by genetic etiology (monogenic vs. non-monogenic) (Fig. 2B). Non-monogenic conditions comprised the majority of cases, though most entries in China’s rare disease catalog are genetic. Monogenic disorders often permit early diagnosis via genetic screening or family history and have clearer gene-targeted therapies; nonetheless, symptomatic and supportive treatments remain the clinical mainstay (Fig. 2C).

### Most prevalent diseases

We then identified the single most common disease within each Orphanet classification (Table 2). In neurologic diseases, generalized myasthenia gravis was most frequent, with ASIRs of 1.532 (95% CI: 0.961–2.316) in 2012 and 2.143 (95% CI: 1.456–3.043) in 2023. ANCA-associated vasculitis led systemic/rheumatic diseases (ASIR 1.078 [95% CI: 0.611–1.762] in 2012; 1.275 [95% CI: 0.761–2.005] in 2023), while malignant melanoma topped neoplastic diseases (ASIR 0.827 [95% CI: 0.427–1.447] in 2012; 0.720 [95% CI: 0.351–1.309] in 2023).

Among the cohort’s five most frequently diagnosed rare diseases—generalized myasthenia gravis, ANCA-associated vasculitis, malignant melanoma, multiple sclerosis, and amyotrophic lateral sclerosis (ALS)—three are neurologic, underscoring the high burden of neurologic disorders in China’s rare disease landscape.

### Sex- and age-related differences

Overall, the male-to-female ratio was 51.58:48.42%. However, certain categories showed marked sex disparities. Female predominance occurred in systemic/rheumatic, neoplastic, ophthalmologic, and bone diseases—for example, ANCA-associated vasculitis, melanoma, retinitis pigmentosa, and McCune-Albright syndrome. Male predominance was seen in neurologic, respiratory, hematologic, circulatory, gastroenterologic, hepatic, cardiac diseases, and inborn errors of metabolism—for example, generalized myasthenia gravis, idiopathic pulmonary fibrosis, paroxysmal nocturnal hemoglobinuria, coronary artery dilation, familial adenomatous polyposis, Wilson’s disease, noncompaction cardiomyopathy, and methylmalonic academia. Renal, skin diseases and developmental defect exhibited near-equal sex distribution (e.g., Gitelman syndrome, postular psoriasis, 21-hydroxylase deficiency).

Age at onset varied by category: most diseases manifested after age 18, reflecting a predominance of late-onset conditions (Fig. 2D). Systemic/rheumatic diseases had the highest mean (67.7 years) and median (69 years) onset ages; idiopathic pulmonary fibrosis, ANCA-associated vasculitis, and melanoma averaged > 60 years. In contrast, embryonic developmental defects, bone diseases, inborn errors of metabolism, and immune diseases had lower onset ages, with pediatric cases (≤ 18 years) comprising 100% of immune diseases, 71.74% of embryonic developmental defects, 80.00% of bone diseases, and 80.89% of metabolic disorders. For example, all X-linked lymphoproliferative disease cases occurred in children, 21-hydroxylase deficiency and McCune-Albright syndrome had 80.00% pediatric onset, and 89.84% of methylmalonic academia were in those ≤ 18 years.

### Global comparison of prevalent rare diseases

To contextualize China’s rare disease burden, we compared five prevalent monogenic diseases (Wilson’s disease, methylmalonic acidemia, phenylketonuria, hemophilia, Gitelman syndrome) and five prevalent non-monogenic diseases (myasthenia gravis, ANCA-associated vasculitis, melanoma, multiple sclerosis, ALS). We calculated the 12-year average incidence rates for these diseases and compared their annual incidence rates from Orphadata across different global regions [16] (Table 3). Significant disparities emerged. For Wilson’s disease, China’s annual incidence was 0.136 per 100,000 (95% CI: 0.016–0.497) versus 0.016 per 100,000 (95% CI: 0.0093–0.026) in Finland—an eight-fold difference. Hemophilia incidence in China was 0.056 per 100,000 (95% CI: 0.001–0.362), compared with 6.25 per 100,000 in Europe (nearly 100-fold higher). Myasthenia gravis incidence was similar to global rates (1.798 per 100,000 [95% CI: 1.174–2.635]), reflecting standardized autoimmune disease diagnostics. ALS incidence in China was 0.342 per 100,000 (95% CI: 0.110–0.803), four to eight times lower than in Europe and North America.

**Table 3:** Rare disease incidence in China and other regions

Rare diseases		12-year average incidence rate in China^*^		Annual incidence rate by region^#^				Reference (PMID/ Orphadata)
				Region		Annual incidence		
Wilson disease		0.136 (0.016, 0.497)		Finland		0.016 (0.009, 0.026)		32,618,023 [31]
Methylmalonic academia		0.084 (0.004, 0.412)		N.A.		N.A.		N.A.
Phenylketonuria		0.052 (0.000, 0.356)		N.A.		N.A.		N.A.
Hemophilia		0.056 (0.001, 0.362)		Europe		6.25		Orphadata
Gitelman syndrome		0.049 (0.000, 0.350)		N.A.		N.A.		N.A.
Generalized myasthenia gravis		1.798 (1.174, 2.635)		Worldwide		0.53 (0.44, 0.61)		20,565,885 [32]
				UK		1.11		9,771,771 [33]
				Spain		2.13 (1.39, 3.12)		12,654,975 [34]
				USA		0.90		8,909,435 [35]
				Australia		2.49		22,469,211 [36]
ANCA-associated vasculitis		1.047 (0.588, 1.724)		N.A.		N.A.		N.A.
Melanoma		0.623 (0.285, 1.182)		N.A.		N.A.		N.A.
Multiple Sclerosis		0.491 (0.199, 1.008)		N.A.		N.A.		N.A.
Amyotrophic lateral sclerosis		0.342 (0.110, 0.803)		Worldwide		1.35		21,665,992 [37]
				Ireland		2.1 (1.8, 2.4)		10,025,778 [38]
				France		2.0 (1.8, 2.3)		19,452,307 [39]
				USA		1.46		25,298,019 [40]

^*^12-year average incidence rate from 2012 to 2023, for all rare diseases are based on the standard population published in the 2023 China Statistical Yearbook, per 100,000 person-years (95%CI)

^#^Annual incidence rates by region sourced from Orphadata, per 100,000 person-years (95%CI)

N.A., Not available

## Discussion

This study offers the first large-scale, population-based assessment of rare diseases in China, leveraging data from 14.31 million the Great Beijing Area residents over 12 years. Our findings elucidate unique epidemiological trends and demographic patterns that both align with and diverge from global observations.

### Rising incidence trends

The overall age-standardized incidence rate (ASIR) of rare diseases in China increased steadily from 6.109 per 100,000 person-years in 2012 to 7.394 in 2023. This upward trajectory likely reflects enhanced disease recognition driven by China’s national rare disease catalogs (2018, 2023) [3, 4]., improved clinician awareness, and the wider availability of advanced diagnostics such as next-generation sequencing and biochemical assays. These policy and technological advances have lowered diagnostic delays and expanded case ascertainment.

### Predominance of neurologic diseases

Rare neurologic diseases emerged as the most common category (52.12% of cases), led by generalized myasthenia gravis, multiple sclerosis, and amyotrophic lateral sclerosis (ALS). This mirrors global data, where neurologic disorders frequently dominate rare disease registries due to their high clinical burden and genetic complexity. Systemic and rheumatologic diseases (16.89%)—particularly ANCA-associated vasculitis—ranked second, contrasting with Western cohorts in which conditions like systemic lupus erythematosus and scleroderma prevail [20, 21]. Such differences may arise from distinct genetic predispositions, environmental exposures, and population-specific immune responses.

### Sex and age disparities

Our study identified notable sex-based disparities in the incidence of rare diseases. Female patients exhibited a higher prevalence of rare systemic and rheumatic diseases, neoplastic diseases, eye conditions, and bone diseases, while male patients were more frequently diagnosed with rare neurologic, respiratory, hematologic, circulatory, gastroenterologic, hepatic, cardiac diseases, and inborn errors of metabolism. These differences are consistent with previous research, which suggests that sex hormones, immune system variations, and genetic susceptibility contribute to sex-specific disease manifestations [22, 23].

Age at diagnosis varied significantly across different disease categories. While many rare diseases were diagnosed in adulthood, conditions such as inborn errors of metabolism and rare immune diseases predominantly affected pediatric patients. Wilson’s disease, a rare autosomal recessive disorder affecting copper metabolism, exhibited a relatively high incidence in China (0.136 per 100,000 population), contrasting sharply with lower rates observed in Nordic countries (0.016 per 100,000 population) [24]. The early-onset nature of this disease underscores the importance of neonatal screening programs to facilitate timely diagnosis and intervention.

### Global comparisons

When compared with Orphadata and other international registries, China’s rare disease incidence exhibits both lower and higher rates depending on the condition. Hemophilia incidence in China (0.056 per 100,000) is nearly 100-fold lower than in Europe (6.25 per 100,000), potentially reflecting underdiagnosis, limited access to specialized care, and regional genetic carrier frequencies [25]. ALS incidence (0.342 per 100,000) is four to eight times lower than in Europe and North America, suggesting genetic or environmental protective factors. Conversely, generalized myasthenia gravis rates are comparable globally, reinforcing the notion of shared autoimmune mechanisms across populations [26].

### Public health implications

Our results carry critical implications for China’s healthcare policy. First, establishing a national rare disease registry is imperative to monitor trends, facilitate research collaboration, and guide resource allocation. Second, expanding newborn and high-risk group screening—particularly for Wilson’s disease and inborn metabolic errors—can enable earlier diagnosis and treatment [27, 28]. Third, equitable access to emerging therapies, including gene therapy and enzyme replacement, must be prioritized within the national health insurance framework. Finally, targeted education for healthcare providers and patient advocacy initiatives will be essential to reduce diagnostic delays and improve quality of life.

### Launching of new drugs for rare diseases in China

The introduction of orphan drugs have significantly advanced rare disease treatment in China, though key challenges remain to ensure their broad and effective use. By the end of 2019, China had approved 83 orphan drugs for the treatment of 29 rare diseases [29]. In recent years, the continuous growth of orphan drug research in China has indicated improved drug accessibility for rare diseases and further promoted the development of a rare disease care system [30]. The introduction of these therapies has transformed rare disease management by offering new options, yet challenges in affordability, access, and long-term monitoring persist. Strengthening regulation, fostering innovation, and ensuring sustainable funding remain essential to fully realize their impact.

### Strengths and limitations

Strengths of this study include its large, population-based design, rigorous case validation via expert committees, and alignment with both national and international classification systems. To enhance case validation, only discharge diagnoses which established by clinicians following comprehensive assessments including medical history, examination findings, and treatment response were included. These diagnoses reflect high clinical certainty and are considered definitive, while interim or provisional diagnoses were excluded to ensure consistency. However, several limitations merit consideration. First, our dataset is restricted to registered residents of the Great Beijing Area, which may potentially underrepresent whole population of China, although Beijing attracts a highly mobile and demographically diverse population, with individuals originating from across both northern and southern China. This internal diversity allows Beijing’s population to approximate, to some extent, the broader ethnic and geographic heterogeneity of the national population of China. Second, reliance on hospital-based diagnoses may lead to under ascertainment of cases, particularly among patients with mild or asymptomatic presentations not requiring hospitalization (e.g., mild biotinidase deficiency), and those with rapidly progressive, high-mortality conditions (e.g., spinal muscular atrophy) who may be missed due to delayed care or misdiagnosis in primary settings. These limitations may introduce systematic bias in incidence estimates. Third, in China, newborn screening (NBS) programs routinely cover conditions like phenylketonuria, other metabolic disorders and hearing loss, with some regions expanding to include rare diseases such as maple syrup urine disease and methylmalonic acidemia. These are often detected early and managed without hospitalization, leading to underrepresentation in hospital-based data and potential underestimation of incidence. Future studies should integrate NBS data with clinical records to improve accuracy in estimating rare disease epidemiology.

## Conclusion

This first comprehensive analysis of rare diseases in China reveals a rising incidence, a predominance of neurologic disorders, and marked demographic and geographic variations. Addressing these challenges will require enhanced diagnostic capabilities, robust registries, equitable treatment access, and tailored public health strategies to improve outcomes for rare disease patients in China.

## Supplementary Information

Below is the link to the electronic supplementary material.

Supplementary Material 1

## Data Availability

Data will be made available on request.
