# Supplementary material for: Spectrum and epidemiology of rare diseases in a Chinese natural population of 14.31 million residents, 2012–2023
Source: Orphanet J Rare Dis. 2025 Aug 7;20:410. doi: 10.1186/s13023-025-03933-8 (PMC12333119; doi:10.1186/s13023-025-03933-8)
Supplement: Supplementary file 1 — Supplementary Material 1 [file 13023_2025_3933_MOESM1_ESM.docx]

**Table S1. Hospitals Included (n=158)**

| 1. | Beijing Baiziwan Harmonic Women and Children's Hospital |
| --- | --- |
| 2. | Beijing North Asia Orthopedic Hospital |
| 3. | Beijing Bo'ai Hospital |
| 4. | Beijing Changping Zhenghe Traditional Chinese Medicine Hospital |
| 5. | Beijing Chaoyang Integrated Traditional Chinese and Western Medicine Emergency Rescue Hospital |
| 6. | Beijing Chengbei Longcheng Hospital |
| 7. | Beijing Dawang Road Emergency Rescue Hospital |
| 8. | Peking University Third Hospital |
| 9. | Peking University First Hospital |
| 10. | Peking University International Hospital |
| 11. | Peking University School of Stomatology |
| 12. | Peking University People's Hospital |
| 13. | Peking University Shougang Hospital |
| 14. | Peking University Hospital |
| 15. | Beijing Fengtai Sanluju Integrated Traditional Chinese and Western Medicine Hospital |
| 16. | Beijing Fengtai Hospital |
| 17. | Beijing Fengtai You'anmen Hospital |
| 18. | Beijing Gaobo Borun Hospital |
| 19. | Beijing Aerospace General Hospital |
| 20. | Beijing Nuclear Industry Hospital |
| 21. | Beijing Huasheng Rehabilitation Hospital |
| 22. | Beijing Huatan Integrated Traditional Chinese and Western Medicine Hospital |
| 23. | Beijing Huaxin Hospital (The First Affiliated Hospital of Tsinghua University) |
| 24. | Beijing Huairou Hospital |
| 25. | Beijing Huilan Hospital |
| 26. | Beijing Jingdu Children's Hospital |
| 27. | Beijing Jingmei Group General Hospital |
| 28. | Beijing Jingxi Tumor Hospital |
| 29. | Beijing Jingxin Hospital |
| 30. | Beijing Jingcheng Bo'ai Hospital |
| 31. | Beijing Geriatric Hospital |
| 32. | Beijing Linke Traditional Chinese Medicine Nephropathy Hospital |
| 33. | Beijing Lu Daopei Hematology Hospital |
| 34. | Beijing Lu Daopei Hospital |
| 35. | Beijing Minzhong Nursing Home |
| 36. | Beijing Nanjiao Tumor Hospital |
| 37. | Beijing Qiangshou Traditional Chinese Medicine Hospital |
| 38. | Beijing Tsinghua Changgung Hospital |
| 39. | Beijing Shizhentang Integrated Traditional Chinese and Western Medicine Hospital |
| 40. | Nankou Railway Hospital, Changping District, Beijing |
| 41. | Nankou Hospital, Changping District, Beijing |
| 42. | Shahe Hospital, Changping District, Beijing |
| 43. | Changping District Hospital, Beijing |
| 44. | Changping District Hospital of Integrated Traditional Chinese and Western Medicine, Beijing |
| 45. | Changping District Hospital of Traditional Chinese Medicine, Beijing |
| 46. | Chaoyang District Maternal and Child Health Hospital, Beijing |
| 47. | Chaoyang District Huanxing Cancer Hospital, Beijing |
| 48. | Shuangqiao Hospital, Chaoyang District, Beijing |
| 49. | Chaoyang District Hospital of Traditional Chinese Medicine, Beijing |
| 50. | Beijing Chuiyangliu Hospital |
| 51. | People's Hospital of Daxing District, Beijing |
| 52. | Daxing District Hospital of Integrated Traditional Chinese and Western Medicine, Beijing |
| 53. | Beijing Second Hospital |
| 54. | Beijing Sixth Hospital |
| 55. | Beijing First Hospital of Integrated Traditional Chinese and Western Medicine |
| 56. | Dongcheng District First People's Hospital, Beijing |
| 57. | Fangshan District First Hospital, Beijing |
| 58. | Liangxiang Hospital, Fangshan District, Beijing |
| 59. | Qiaoliang Factory Workers' Hospital, Fangshan District, Beijing |
| 60. | Fangshan District Hospital of Traditional Chinese Medicine (Fangshan Hospital of Beijing University of Chinese Medicine), Beijing |
| 61. | Fengtai Rehabilitation Hospital, Beijing |
| 62. | Fengtai District Hospital of Traditional Chinese Medicine, Beijing |
| 63. | Fengtai Hospital of Integrated Traditional Chinese and Western Medicine, Beijing |
| 64. | Beijing Anorectal Hospital |
| 65. | Haidian Hospital, Beijing |
| 66. | Hepingli Hospital, Beijing |
| 67. | Beijing Red Cross Emergency Medical Center (Beijing Red Cross Trauma Hospital) |
| 68. | Huairou District Hospital of Traditional Chinese Medicine, Beijing |
| 69. | Beijing Hui Min Hospital |
| 70. | Beijing Prison Administration Central Hospital |
| 71. | Beijing Jianguo Hospital |
| 72. | Beijing Longfu Hospital (Beijing Geriatric Hospital of Integrated Traditional Chinese and Western Medicine) |
| 73. | Mentougou District Hospital, Beijing |
| 74. | Mentougou District Hospital of Traditional Chinese Medicine (Mentougou District Geriatric Hospital), Beijing |
| 75. | Miyun District Hospital, Beijing |
| 76. | Miyun District Hospital of Traditional Chinese Medicine (Miyun Branch of the Third Affiliated Hospital of Beijing University of Chinese Medicine), Beijing |
| 77. | Pinggu District Hospital, Beijing |
| 78. | Pinggu District Hospital of Traditional Chinese Medicine, Beijing |
| 79. | Pinggu Yuexie Hospital, Beijing |
| 80. | Beijing Purren Hospital |
| 81. | Beijing Renhe Hospital |
| 82. | Beijing Social Welfare Hospital |
| 83. | Beijing Shijingshan Hospital |
| 84. | Shunyi District Maternal and Child Health Hospital (Shunyi Women and Children's Hospital of Beijing Children's Hospital) (Shunyi District Maternal and Child Health and Family Planning Service Center), Beijing |
| 85. | Shunyi District Airport Hospital (Houshayu Community Health Service Center of Shunyi District), Beijing |
| 86. | Shunyi District Hospital, Beijing |
| 87. | Shunyi District Hospital of Traditional Chinese Medicine (Shunyi Hospital of Beijing Hospital of Traditional Chinese Medicine), Beijing |
| 88. | Tongzhou District Maternal and Child Health Hospital, Beijing |
| 89. | Tongzhou District Hospital of Integrated Traditional Chinese and Western Medicine, Beijing |
| 90. | Tongzhou District Hospital of Traditional Chinese Medicine, Beijing |
| 91. | Guangwai Hospital of Xicheng District (Guangwai Geriatric Hospital of Xicheng District), Beijing |
| 92. | Ping'an Hospital of Xicheng District, Beijing |
| 93. | Zhanlanlu Hospital of Xicheng District, Beijing |
| 94. | Xuanwu Hospital of Traditional Chinese Medicine, Beijing |
| 95. | Yanqing District Hospital (Yanqing Hospital of Peking University Third Hospital), Beijing |
| 96. | Beijing Yangfangdian Hospital |
| 97. | Beijing Zhongguancun Hospital (Zhongguancun Hospital of Chinese Academy of Sciences) |
| 98. | Beijing Water Resources Hospital |
| 99. | Beijing Sihui Hospital of Traditional Chinese Medicine |
| 100. | Beijing Sijiqing Hospital |
| 101. | Beijing Tongji Dongfang Hospital of Integrated Traditional Chinese and Western Medicine |
| 102. | Beijing Tongrentang Hospital of Traditional Chinese Medicine |
| 103. | Beijing Wangfu Hospital of Integrated Traditional Chinese and Western Medicine |
| 104. | Beijing Xiaotangshan Hospital |
| 105. | Beijing New Century Children's Hospital |
| 106. | Beijing Yanhua Hospital |
| 107. | Beijing Yaoyi Hospital |
| 108. | Beijing Hospital |
| 109. | Beijing Yuhe Hospital of Integrated Traditional Chinese and Western Medicine Rehabilitation |
| 110. | Beijing Changfeng Hospital |
| 111. | Beijing Hospital of Integrated Traditional Chinese and Western Medicine |
| 112. | Beijing Zhongyan Group Dongcheng Hospital of Traditional Chinese Medicine |
| 113. | Third Affiliated Hospital of Beijing University of Chinese Medicine |
| 114. | Dongfang Hospital of Beijing University of Chinese Medicine |
| 115. | Dongzhimen Hospital of Beijing University of Chinese Medicine |
| 116. | Huguo Temple Hospital of Traditional Chinese Medicine Affiliated to Beijing University of Chinese Medicine |
| 117. | Yanqing Hospital of Beijing Hospital of Traditional Chinese Medicine (Yanqing District Hospital of Traditional Chinese Medicine), Beijing |
| 118. | Beijing Cancer Hospital |
| 119. | State Grid Beijing Electric Power Hospital |
| 120. | Rehabilitation Hospital Affiliated to the National Research Center for Rehabilitation Technical Aids |
| 121. | Aviation General Hospital |
| 122. | Aerospace Center Hospital |
| 123. | Civil Aviation General Hospital |
| 124. | Tsinghua University Hospital |
| 125. | Tsinghua University Yuquan Hospital (Tsinghua University Hospital of Integrated Traditional Chinese and Western Medicine) |
| 126. | Children's Hospital Affiliated to the Capital Institute of Pediatrics |
| 127. | Emergency Medical Rescue Center of Capital Airports Holding Company |
| 128. | Beijing Anzhen Hospital Affiliated to Capital Medical University |
| 129. | Beijing Chaoyang Hospital Affiliated to Capital Medical University |
| 130. | Beijing Ditan Hospital Affiliated to Capital Medical University |
| 131. | Beijing Children's Hospital Affiliated to Capital Medical University |
| 132. | Beijing Obstetrics and Gynecology Hospital Affiliated to Capital Medical University |
| 133. | Beijing Jishuitan Hospital Affiliated to Capital Medical University |
| 134. | Beijing Rehabilitation Hospital Affiliated to Capital Medical University (Beijing Workers' Sanatorium) |
| 135. | Beijing Luhe Hospital Affiliated to Capital Medical University |
| 136. | Beijing Shijitan Hospital Affiliated to Capital Medical University (Beijing Railway General Hospital) |
| 137. | Beijing Tiantan Hospital Affiliated to Capital Medical University |
| 138. | Beijing Tongren Hospital Affiliated to Capital Medical University |
| 139. | Beijing Chest Hospital Affiliated to Capital Medical University |
| 140. | Beijing Friendship Hospital Affiliated to Capital Medical University |
| 141. | Beijing Friendship Hospital (Tongzhou Branch) Affiliated to Capital Medical University |
| 142. | Beijing You'an Hospital Affiliated to Capital Medical University |
| 143. | Beijing Hospital of Traditional Chinese Medicine Affiliated to Capital Medical University |
| 144. | Fuxing Hospital Affiliated to Capital Medical University |
| 145. | Sanbo Brain Hospital Affiliated to Capital Medical University |
| 146. | Xuanwu Hospital Affiliated to Capital Medical University |
| 147. | Emergency General Hospital of the Ministry of Emergency Management |
| 148. | Beijing Tibetan Hospital of China Tibetology Research Center |
| 149. | 731 Hospital of China Aerospace Science and Industry Corporation |
| 150. | Peking Union Medical College Hospital of the Chinese Academy of Medical Sciences |
| 151. | Fuwai Hospital of the Chinese Academy of Medical Sciences |
| 152. | Cancer Hospital of the Chinese Academy of Medical Sciences |
| 153. | Guang'anmen Hospital of China Academy of Chinese Medical Sciences |
| 154. | Guang'anmen Hospital (Southern District) of China Academy of Chinese Medical Sciences |
| 155. | Wangjing Hospital of China Academy of Chinese Medical Sciences |
| 156. | Xiyuan Hospital of China Academy of Chinese Medical Sciences |
| 157. | Eye Hospital of China Academy of Chinese Medical Sciences |
| 158. | China-Japan Friendship Hospital |

**Table S2. Orphanet-based linear classification of rare diseases in dataset**

| Orphanet classification | Name or group of diseases divided according to China’s national rare disease lists (2018 and 2023) | Examples of diseases included in groups |
| --- | --- | --- |
| Rare neurologic disease | Generalized myasthenia gravis | Myasthenia gravis |
|  |  | Mild generalized myasthenia gravis |
|  |  | Moderate generalized myasthenia gravis |
|  |  | Acute severe myasthenia gravis |
|  |  | Late-onset severe myasthenia gravis |
|  |  | Myasthenic crisis |
|  |  | Juvenile myasthenia gravis |
|  | Progressive muscular dystrophy | Muscular dystrophy |
|  |  | Duchenne muscular dystrophy |
|  |  | Becker muscular dystrophy |
|  |  | Emery-dreifuss muscular dystrophy |
|  |  | Progressive muscular dystrophy |
|  |  | Oculopharyngeal muscular dystrophy |
|  |  | Pseudohypertrophic muscular dystrophy |
|  |  | Distal muscular dystrophy |
|  |  | Facioscapulohumeral dystrophy |
|  |  | Limb-girdle muscular dystrophy |
|  | Multiple sclerosis | Multiple sclerosis |
|  |  | Relapsing-remitting multiple sclerosis |
|  |  | Primary progressive multiple sclerosis |
|  |  | Secondary progressive multiple sclerosis |
|  |  | Progressive-relapsing multiple sclerosis |
|  |  | Clinically isolated syndrome |
|  | Spinocerebellar ataxia | Spinocerebellar ataxia |
|  |  | X-linked recessive spinocerebellar ataxia |
|  | Amyotrophic lateral sclerosis | Hereditary ataxia |
|  |  | Motor neuron disease |
|  |  | Amyotrophic lateral sclerosis |
|  |  | Progressive spinal muscular atrophy |
|  | Primary hereditary dystonia | Familial motor neuron disease |
|  |  | Idiopathic dystonia |
|  |  | Idiopathic familial dystonia |
|  |  | Meige syndrome |
|  |  | Focal dystonia |
|  | Spinal muscular atrophy | Generalized dystonia |
|  |  | Adult-onset spinal muscular atrophy type IV |
|  |  | Infantile spinal muscular atrophy type II |
|  |  | Distal spinal muscular atrophy |
|  | Spinal and bulbar muscular atrophy (Kennedy disease) | Kennedy disease |
|  | Neuromyelitis optica | neuromyelitis optica |
|  |  | neuromyelitis optica spectrum disorders |
|  | Multiple system atrophy | Multiple system atrophy |
|  | Myotonic dystrophy | atrophic myotonia |
|  |  | dystrophia myotonia |
|  | Multifocal motor neuropathy | Multifocal motor neuropathy |
|  | Lennox-Gastaut syndrome | Lennox-Gastaut syndrome |
|  | Charcot-Marie-Tooth disease | Charoneal muscle atrophy |
|  | Congenital myasthenic Syndrome | Congenital myasthenic syndrome |
|  |  | congenital myasthenia gravis |
|  | Congenital myotonia syndrome (Non-Dystrophic Myotonia, NDM) | Congenital myotonia |
|  | Autoimmune encephalitis | NMDA receptor encephalitis |
|  |  | Autoimmune encephalitis |
|  | Mitochodrial encephalomyopathy | Mitochondrial encephalomyopathy with lactic acidosis and stroke-like episodes (MELAS) |
|  | CDKL5-deficiency disorder | Early myoclonic encephalopathy |
|  | West syndrome/Infantile spasms syndrome | West syndrome/Infantile spasms syndrome |
| Rare systemic and rheumatologic disease | ANCA-associated vasculitis | ANCA-associated vasculitis |
|  | Erdheim-Chester Disease | Erdheim-Chester Disease |
|  | IgG4 related Disease | IgG4 related Disease |
|  | Familial mediterranean fever | Familial mediterranean fever |
|  | Langerhans cell histiocytosis | Langerhans cell histiocytosis, unifocal disease |
|  | Systemic sclerosis | Progressive systemic sclerosis |
|  |  | Systemic sclerosis-associated interstitial lung disease |
|  | Primary light chain amyloidosis | primary amyloidosis |
|  |  | primary systemic amyloidosis |
|  | Cryopyrin associated periodic syndrome/ NLRP3-associated systemic autoinflammatory disease | Cold urticaria |
| Rare neoplastic disease | Malignant melanoma | Malignant melanoma of the eyelid |
|  |  | Malignant melanoma of the ear |
|  |  | Malignant melanoma of the face |
|  |  | Malignant melanoma of the nose |
|  |  | Malignant melanoma of the neck |
|  |  | Malignant melanoma of the trunk |
|  |  | Malignant melanoma of the breast |
|  |  | Malignant melanoma of the abdominal wall |
|  |  | Malignant melanoma of the inguinal region |
|  |  | Malignant melanoma of the back |
|  |  | Malignant melanoma of the buttock |
|  |  | Malignant melanoma of the anus |
|  |  | Malignant melanoma of the upper limb |
|  |  | Malignant melanoma of the hand |
|  |  | Malignant melanoma of the lower limb |
|  |  | Melanoma in situ of the trunk |
|  |  | Melanoma in situ of the anal skin |
|  |  | Melanoma in situ of the perianal area |
|  |  | Melanoma in situ of the upper limb |
|  |  | Melanoma in situ of the lower limb |
|  | Multiple endocrine neoplasia | Multiple endocrine neoplasia |
| Rare respiratory disease | Pulmonary cystic fibrosis | Pulmonary Cystic Fibrosis |
|  |  | Cystic fibrosis with pulmonary manifestations |
|  |  | Pulmonary alveolar proteinosis |
|  | Idiopathic pulmonary fibrosis | Hamman-Rich syndrome |
|  |  | Idiopathic pulmonary fibrosis |
|  | Idiopathic pulmonary arterial hypertension | Heritable pulmonary arterial hypertension |
|  |  | Persistent pulmonary hypertension of the newborn |
|  |  | Idiopathic pulmonary arterial hypertension |
| Rare eye disease | Retinitis pigmentosa | Retinitis pigmentosa |
| Rare hematologic disease | Atypical hemolytic uremic syndrome | Hemolytic uremic syndrome |
|  | Paroxysmal nocturnal hemoglobinuria | paroxysmal nocturnal hemoglobinuria |
|  | Hemophilia | Hemophilia |
|  |  | Hemophilia A |
|  |  | Hemophilia B |
|  | Thrombotic thrombocytopenic purpura | Thrombotic thrombocytopenic purpura |
|  | Thalassemia major | Thalassemia major |
| Inborn errors of metabolism | Hyperphenylalaninemia | phenylketonuria |
|  | Propionic acidemia | Propionic acidemia |
|  | Methylmalonic academia | methylmalonic acidemia |
|  | Porphyria | Porphyria |
|  | Maple syrup urine disease | Maple syrup urine disease |
|  | Gangliosidosis | GM3 gangliosidosis |
|  | Fabry disease | Fabry disease |
|  | Gaucher’s disease | Gaucher’s disease |
|  | Niemann-Pick disease | Niemann-Pick disease |
|  | Very long chain Acyl-CoA dehydrogenase deficiency | Very long chain Acyl-CoA dehydrogenase deficiency |
|  | Glycogen Storage Disease | Glycogen Storage Disease |
|  | Mucopolysaccharidosis | Mucopolysaccharidosis type III |
|  | X-linked adrenoleukodystrophy | X-linked adrenoleukodystrophy |
| Rare circulatory system disease | Coronary artery ectasia | Coronary artery ectasia |
| Rare gastroenterologic disease | Short bowel syndrome | Short bowel syndrome |
|  | Eosinophilic gastroenteritis | Eosinophilic gastroenteritis |
|  | Familial adenomatous polyposis | Familial adenomatous polyposis |
| Rare hepatic disease | Hepatolenticular degeneration (Wilson disease) | Wilson disease |
| Rare cardiac disease | idiopathic cardiomyopathy | Idiopathic cardiomyopathy |
|  |  | Restrictive cardiomyopathy |
|  |  | Noncompaction of the ventricular myocardium |
|  |  | Right ventricular cardiomyopathy |
|  | Cardiac ion channelopathies | Congenital long QT syndrome |
| Rare renal disease | Gitelman syndrome | Gitelman syndrome |
| Rare developmental defect during embryogenesis | Congenital scoliosis | Congenital hemivertebra deformity |
|  | Fanconi anemia | Fanconi anemia |
|  | 21-hydroxylase deficiency | 21-hydroxylase deficiency |
| Rare bone disease | McCune-Albright syndrome | McCune-Albright syndrome |
| Rare skin disease | Generalized pustular psoriasis | Pustular psoriasis |
| Rare endocrine disease | Homozygous hypercholesterolemia | Homozygous familial hypercholesterolemia |
|  | Acromegaly | Acromegaly |
| Rare immune disease | Severe congenital neutropenia | congenital neutropenia |
|  | X-linked lymphoproliferative disease | X-linked lymphoproliferative disease |
